# Large-scale implementation of electronic Integrated Management of Childhood Illness (eIMCI) at the primary care level in Burkina Faso: a qualitative study on health worker perception of its medical content, usability and impact on antibiotic prescription and resistance

**DOI:** 10.1186/s12889-019-6692-6

**Published:** 2019-04-29

**Authors:** Cécile Bessat, Noël Adannou Zonon, Valérie D’Acremont

**Affiliations:** 1 Department of Ambulatory Care and Community Medicine, Lausanne, Switzerland; 2 University of Lausanne Medical School, Lausanne, Switzerland; 3 Terre des hommes, Ouagadougou, Burkina Faso; 4 Swiss Tropical and Public Health Institute, University of Basel, Lausanne, Switzerland

**Keywords:** Computerized decision support system, Electronic clinical algorithms, Integrated management of childhood illness, Tablets, Antibiotic use, Antimicrobial resistance, Diagnostic tools, Low-resource countries, Primary care, Clinician perception

## Abstract

**Background:**

Electronic clinical decision algorithms (eCDAs) that guide clinicians during patient management are being deployed in resource-limited settings to improve the quality of care and rational use of medicines (especially antimicrobials). Little is known on how local clinicians perceive the use and impact of these tools in their daily practice. This study investigates clinician insights on an eIMCI tool. Specifically, we report their views on its medical content, assess their knowledge on microbes, antimicrobials and the development of resistance.

**Methods:**

This qualitative study was conducted in the frame of a large-scale implementation in Burkina Faso of an eIMCI tool developed by the Swiss NGO Terre des hommes. Twelve in-depth interviews and 2 focus-group discussions were conducted including 21 health workers from 10 primary care facilities. Emerging themes were identified using qualitative data analysis software.

**Results:**

eIMCI users expressed a high level of satisfaction, slowness of the tablet was perceived as the major inconvenience limiting uptake. Several frequent illnesses were identified as missing in the algorithm along with guidance for fever without focus. When asked about existing types of microbes, 9 and 4 out of 21 participants could mention bacteria and virus respectively; only 5 correctly answered that antibiotics had no action on viral disease and 6 mentioned the risk of antibiotic resistance. Level of knowledge was higher in nurses than in less trained health workers. The tool was perceived as improving patient management and the rational use of antibiotics. Positive changes in health facility organisation were reported, such as task shifting and improved triage. eIMCI was also perceived as a learning tool, and users expressed a strong desire to expand the geographic and temporal scope of the intervention.

**Conclusion:**

The use of eICMI was widely accepted and perceived as a powerful tool guiding daily practice. Findings suggest that it has positive effects on the health care system beyond the quality of consultation. To support large uptake and sustainability, better training of health workers in infectiology is essential and the medical content of eIMCI should be optimized to include frequent diseases and, for each of them, the appropriate management plan.

**Electronic supplementary material:**

The online version of this article (10.1186/s12889-019-6692-6) contains supplementary material, which is available to authorized users.

## Background

Since 1990, global child mortality has more than halved. Despite this substantial achievement, it falls short of the 2015 Millennium Development Goal of a two thirds reduction, and remains a problem that disproportionately affects Sub-Saharan Africa [1]. So far, the reductions have been made possible by global immunization campaigns, coordinated malaria control programs and standardized clinical guidelines for management of childhood disease. The most widely used clinical guideline is the “Integrated Management of Childhood Illness” (IMCI), which was developed in the mid-nineties by the World Health Organisation (WHO) and the United Nations International Children’s Emergency Fund (UNICEF) in order to improve the management of common childhood illnesses at the primary health care level by enabling early diagnosis and effective treatment.

When correctly applied, the IMCI strategy has shown the potential to significantly reduce children morbidity and mortality [2]. Its real impact over the years has however remained less than expected due to limited uptake and poor adherence to its guidelines, frequently resulting in incorrect classification and treatment [3]. In a study undertaken in Burkina Faso in 2013, only 11% of children were assessed for the four key danger signs. While 66% were correctly classified, just 11% received prescriptions according of IMCI recommendations [4]. Many contributing factors have been identified for these poor results, including insufficient training, shortage of staff, lack of supervision, and work overload [5, 6]. Despite great efforts to increase training and supervision, the rate of IMCI adherence has remained very low [7]. Moreover, users have reported to find the IMCI chart booklet heavy and tedious [8].

With the recent advances and expansion of access to new technologies, the use of electronic clinical decision algorithms (eCDAs) in low-income countries have become feasible and hold the potential to overcome those challenges and improve the management of childhood illnesses. eCDAs are currently being developed and deployed in resource-limited countries with the aim to increase the clinician adherence to health recommendations and therefore improve the quality of care.

In a pilot study conducted in Tanzania, the use of an electronic version of IMCI (eIMCI) on personal digital assistants (PDAs) has shown to increase adherence to IMCI guidelines, reduce errors and improve the quality of care in the same consultation time as routine practice [9]. In another study, mobile technologies have improved communication with the caretaker and the impact of counselling [10]. Further, health workers reported that the management of children with eIMCI on PDAs was faster and easier, and encouraged adherence to IMCI guidelines [11]. Shao et al. identified several influencing factors for long term uptake and adherence; barriers cited were lack of staff, medicine and financial incentive, while facilitators were training, supervision and long-term follow-up [12].

Non-adherence to clinical guidelines not only impacts individual morbidity and mortality, but also results in the over prescription of antimicrobials which contributes to global antimicrobial resistance. Interventions guiding rational use of antimicrobials by first line health care providers are urgently needed. In two studies in Tanzania, an electronic IMCI-derived algorithm called ALMANACH, as well as a new algorithm connected to point-of-care tests called e-POCT, have shown to improve clinical outcome and reduce antibiotic prescription by more than 80% [13, 14]. Interventions using eCDAs have the potential to rationalize the use of antimicrobials and consequently mitigate the development of resistance. This, however, is only possible if clinicians understand the difference between a bacterium and a virus, the action of antibiotics and the risk of development of resistance in order to build knowledge-based trust in the prescription recommendations given by the eCDA.

Little is known about the health workers’ opinion on the use of an electronic tool to support decision-making in their daily practice. This study investigates the clinician’s perspective at the primary care level, specifically the acceptability of the tool and their opinion on its medical content. We also assess health worker knowledge on microbes, the role of antimicrobials and the development of antibiotic resistance.

## Methods

### Study design and setting

At the time of the present study, the IeDA intervention was deployed in 8 districts across 2 regions of Burkina Faso (Region Nord and Boucle du Mouhoun) including a total of 272 primary health care facilities (centre de santé et de prévention sociale: CSPS’s) and 1262 primary health care workers using the electronic algorithm, covering a population of around 500,000 children under 5-years-old.

The interviews were conducted in CSPS’s of rural areas of the district of Yako and Toma between January and February 2017. A total of 10 health facilities (HF) were chosen among those using REC. HF were chosen by convenience regardless their size or affluence. In order to have a sample as representative as possible, we chose HF with different levels of uptake and compliance to the electronic recommendations, according to the data collected by supervisors and coordinators from the district team and Tdh.

More information about the study context such as Burkina Faso’s health profile and Burkina Faso’s health system organization is available in the Additional file 1.

### The IeDA intervention

This study was conducted in the frame of the Integrated electronic Diagnostic Approach (IeDA) project in Burkina Faso. This project developed by a Swiss-based NGO called Terre des hommes (Tdh), aims to enhance the application of IMCI guidelines, through the use of an electronic tool guiding clinicians in the assessment, classification and treatment of children under the age of 5 years. For that purpose, Tdh has been working since 2010 on the development and deployment of the REC (registre électronique des consultations), an electronic algorithm based on a version of IMCI from Burkina Faso. The REC application requires that clinicians answer a series of questions related to the clinical presentation of the child (vital signs, clinical signs and symptoms) as well as perform a malaria rapid diagnostic test (RDT). It then displays the list of all diagnostic classifications that need to be considered in parallel, as well as the corresponding treatments, management strategies (in particular the need to refer the child to a higher level of care) and advice to be given to the caregivers, including the indication to reconsult. During each consultation individualized reports are generated by the mobile application and saved into the device (tablet) consultations data is synchronized with the server over mobile network at least on a weekly basis.

### Participant selection and data collection

Participants were selected among workers available in the health facility at the time of the study, trying to cover the widest range of the type of professional using REC. A series of 12 in-depths interviews (IDI) and 2 focus group discussions (FGD) including 4 and 5 participants respectively were conducted in French (the local language) by the first author (no relationship with either Tdh or local health authorities). Interviews were conducted using a semi-structured interview guide developed for this study. The questionnaire included about 20 topics, organized into 6 sections: 1) confidence in the electronic device and technical issues 2) structure and medical content of the algorithm, 3) trust in and compliance to the recommendations given by the electronic device, 4) knowledge on the use of antimicrobial medicines and resistance, 5) perception on IMCI and REC training and supervision, 6) personal experience and suggestion on improvement for future deployment. For each question, probes were used to generate further explanations from study participants. Each interview was conducted as an informal discussion with open questions, leaving room for the health worker to express additional elements. In order to reduce study bias, participants were strongly encouraged to be as open as possible in expressing their opinion and reassured on full confidentiality, especially since research team conducting the interviews had no relationship with Tdh or health authorities.

### Data analysis

All IDI and FGD were audio-recorded with the participant’s informed consent. Detailed notes were taken during the interviews. Records were manually transcribed in its original language (French). A qualitative data analysis software (MAXQDA12) was used to code the French interview transcripts in order to identify emerging themes. A predefined list of codes based on the interview guide and the recurrent themes highlighted in the detailed notes was used. New codes were created when new themes were identified during more detailed analysis of the transcripts. Themes were sorted into 8 different dimensions: 1) Human-electronic interface, 2) Electronic and other technical issues, 3) Medical content of the algorithm, 4) Medical knowledge on microbes and antimicrobials, 5) Impact of the use of REC on patient management, 6) Influence of the use of REC on consultation organisation and workflow at the health facility, 7) Training and supervision provided by the district and 8) Proposed improvement for future implementation.

## Results

### Characteristics of the study participants

A total of 21 health workers were interviewed: 12 during IDI and 9 during FGD. Socio-demographic characteristics of the study participants are described in Table 1. Regardless of their age, all participants had basic IT knowledge before the implementation of the REC.

**Table 1:** Characteristics of the study participants

Characteristic	Number of HW N(%) *N* = 21
Sex (Female)	13 (62)
Age	
- 30–39 years	14 (67)
- 40–49 years	6 (29)
- 50–59 years	1 (5)
Professions^a^	
- Health officer (HO)	6 (29)
- Birth attendant (BA)	7 (33)
- Midwife (MW)	1 (5)
- Licensed nurse (LN)	1 (5)
- Registered nurse (RN)	6 (29)
IMCI and/or REC training	
- None	2 (10)
- REC 2 days	2 (10)
- IMCI 6 days + REC 2 days	6 (29)
- IMCI 11 days + REC 2 days	9 (43)
- Missing data	2 (10)
Electronic knowledge	21 (100.0)
- Personal use of smartphone	14 (67)
- Tablets	0 (0.0)
- Computer	1 (5)
- None	1 (5)
- Missing data	5 (24)
Duration of REC use	
- Since 2010	1 (5)
- Since 2013	1 (5)
- Since 2014	4 (19)
- Since 2015	8 (38)
- Since 2016	1 (5)
- Missing data	6 (29)

^a^Number of years of education required for these professions (in addition to the 6 years of primary school):

Health officer (HO): 2 years of training on primary health care

Birth attendant (BA): 2 years of training on maternal health

Midwife: 4 years of secondary school and 2 years of training on curative care

Licensed practical nurse (LN): 4 years of secondary school and 3 years of training on maternal health

Registered nurse (RN): 4 years of secondary school and 3 years of training on curative care

### Dimension 1: human-electronic interface

#### Sense of confidence

All participants felt confident and comfortable when using the electronic device with patients during consultations. They referred to it as “a help”, “a guide” and “a complete tool”. They mentioned the pride of using it, feeling that it increased HF credibility and the patient’s trust in the delivered care. In one FGD, comfort with the electronic device appeared to be reduced in the rainy season, due however to the high frequentation of the HF and the shorter time available to do the consultation, rather than the use of the electronic device itself.

#### Completeness of the tool

Nearly all health workers (11 IDI, 2FGD) mentioned that the electronic device was simplifying their daily work. They referred to it very often as a complete tool guiding them through all steps of the child’s assessment. Most frequent reasons for that were: support for treatment decision and medicine dose calculation (7IDI, 1FGD); all tools for children assessment and management gathered in a single device (6IDI); easier than the paper version (4IDI, 1FGD); enables systematic malnutrition screening, follow up and alimentation counselling (4IDI); no risk to neglect a step in the assessment (1IDI, 1FGD); help in deciding about the level of disease severity through assessment of danger signs (1IDI).

#### Technology as a source of motivation

Some health workers (1IDI, 1FGD) mentioned that using an electronic device for consultations was a source of motivation, as they felt more advanced compared to other HFs. Some health workers (4IDI, 1FGD) expressed that they perceived automatic data record through the tablet as an advantage, facilitating follow up of patients (3IDI) but also enabling the generation of more accurate statistical analysis (1IDI, 1FGD).

#### Patients’ opinion as reported by health workers

Participants (7IDI) reported the general satisfaction of families regarding the IeDA intervention, due to better confidence in the management and treatment of the child and in health worker’s work in general. Some families even expressed their desire that their child would “enter” in the electronic tool. Some participants (5IDI) reported that the caretakers were expressing dissatisfaction about slowness of consultation and longer waiting times when the REC was used. However, several clinicians (3IDI) countered that those complaints emerged at the beginning of tablet implementation, mainly due to the caretaker’s belief that health workers were playing with their mobile device. They posited that consequently the complaints disappeared with time and explanations. In our informal discussions with the villagers, one mentioned that, during consultations, their children were “touched by nurses much more often than before” and that they were happy about that. One village leader summarized the perception of the community of the “magic tablet” (as they called it) by saying that this tool “brought light where before there was darkness”, referring probably to the increased transparency in patient management and prescription of medicines. He also said that they were ready to buy the tablet with village funds for the sustainability of the project if necessary (Table 2).

**Table 2:** Citations by participants related to dimension 1

Dimension 1: Human-electronic interface	
Sense of confidence	
*“I feel very confident because this tool [REC] gives our service even more credibility. (…) When you go to a health facility in this environment, it is rare to find that your child will be managed with an electronic device. People are perfectly aligned with us on its use and really trust in this device. (…) Not only we are confident and happy to use REC but the patient and caretakers are really assured because they know their child will benefit from a much more appropriate treatment” RN, male*	
Completeness of the tool	
*“I am very happy using REC because it helps me, it guides me through all the childhood illnesses. For example, if you just ask the questions [provided by REC], the disease that the child is suffering from and the treatment needed comes out at the same time, you don’t need to look any further for which treatment to administer to the child. Everything is included in REC. And if a child comes back for a second consultation, you can find them easily, and don’t need to register them a second time” Birth attendant, female*	
Technology as a source of motivation	
*“With the paper version, there was not too much motivation. I remember being trained on the paper version (…) we started to use it but in the uptake evaluation undertaken soon after, one realized that people were actually not using it, they gave up on the paper version. (…) But with this electronic version [REC], we are ahead” RN, male*	*“We receive statistics of our and other health facilities, to see our [compliance] level. It creates a competitive system. It’s this that provides incentive. For example, [you are motivated] when you see that your application rate is good compared to other health facilities.” FGD*
*“When a child comes back, you find them easily [in REC].” Birth attendant, female*	
Patients’ opinion as reported by health workers	
*“Before, mothers were complaining. But with the explanations we gave them, we got them to understand, we sensitized them, we informed them that they would benefit from REC, that the treatments would be more appropriate than before, that if we take care of their child with the device [REC], that we have a better chance to more rapidly detect what their child is suffering from. (...) Mothers talk to each other, and then they ask you why you don’t apply REC to all [ages of] children” E9 HO, Female*	*“The mothers understand when they come to the health facility, none complain. On the contrary, if you start the consultation writing in the [paper] register, the mother tends to give you a perplexed look and asks why the other children got the tablets and not her child” Birth attendant, female*

### Dimension 2: electronic and other technical issues

#### Slowness and breakdowns

A barrier that emerged from the interviews was the “slowness” of the device (mentioned by all participants, and overall 35 times in the interviews), which was perceived as an inconvenience as it increased the duration of consultation. In high-attendance periods, slowness was identified as a source of complaint from the children’s guardians or other patients as it created longer waiting times. Even if most of the participants reported not to be influenced in their compliance to the REC recommendations even in this situation, slowness was clearly reported as a factor endangering uptake in high-attendance periods.

Participants reported some breakdowns requiring a quick restart of the electronic device. Bigger breakdowns that could deprive a primary health facility of REC for several days were reported to be very rare.

#### Additional workload

Several health workers (3IDI, 1FGD) cited barriers to uptake relating to increased workload and the perception of double work when they have to fill the monthly activity register. The data generated automatically by the REC cannot yet be used for the monitoring and surveillance activities of the Ministry of Health. Therefore, the staff remains compelled to fill in the register manually, reporting all the consultations with diagnoses and treatments provided. For children under 5, the work is then double; moreover, the diagnostic classifications provided by the REC often do not correspond to those listed in the register, causing problems for transcription.

Some participants (3IDI, 1FGD) named relative disadvantages of the use of an electronic algorithm to manage children such as the fear of data loss (1IDI, 1FGD), uselessness in case of power outages or breakdowns (1IDI), and impossibility of skipping steps (1IDI).

#### Consultation duration

Regarding the relative duration of consultations with the REC compared to the IMCI paper version, opinions were divergent. Some health workers (7IDI, 2FGD) reported a relative increase in time of consultation; main reasons mentioned were slowness and length of the algorithm. Other participants (4IDI, 2FGD) related on the contrary a decrease in consultation duration. In 4 interviews (2IDI, 2FGD) participants reported both an increase and a decrease during the same conversation. When asked more precisely in the FGD, they concluded that the REC reduces the consultation time compared to a properly led IMCI paper consultation but that it increased it compared to routine practice without IMCI (Table 3).

**Table 3:** Citations by participants related to dimension 2

Dimension 2: Electronic and other technical issues	
Slowness and breakdowns	
*“Sometimes you are alone in front of a sea of patients. When REC is slow, people wait outside, they don’t understand the use and importance of REC, they assume we are playing with our mobile phones and they complain. (…) So we explain to them and little by little they understand. We do our best to register all children so that they aren’t lost to the REC [database], we consult and wait for REC to start working again, and then we continue.” HO, Female.*	
Other electronic issues	
*“If there is a power outage or if the device breaks down, we cannot work. Therefore, to maintain functionality, it is necessary that someone takes close care of maintenance because devices can be very fragile” RN male*	*“With the paper version, the advantage is that you can skip some parts and come back to them afterwards, with REC you are obligated to follow all the steps in order until the end” RN male*

### Dimension 3: medical content of the algorithm

#### Language or miscomprehension

Despite the relative clarity of the medical content of the algorithm, explanations to the child’s guardian was reported by some health workers to be a challenge (3 IDI) due to the different language used by the caregiver or because of the guardian’s understanding. Difficult expressions pointed out were: “presence of blood in stool” (1 IDI), the danger sign “vomits everything” (1 IDI) and “convulsion” (2 IDI).

#### Missing pathologies and absence of final diagnosis or treatment

All health workers mentioned that the REC algorithm was lacking recommendations for the management of frequent diseases. Most frequent missing diseases or health problems mentioned were: burns and wounds (7 IDI, 1 FGD), dermatitis (5 IDI, 2 FGD), digestive candidiasis (6 IDI), urinary tract infection (5 IDI), rhinitis (3IDI, 1FGD) and conjunctivitis (3IDI). At the end of the assessment of the child, the REC application summarizes all the diagnostic classifications obtained. Very often, they correspond to “negative classifications” such as for example: “Fever: no malaria” or “Diarrhoea: no dehydration”. Those negative classifications and relative lack of final diagnosis were reported to be unclear by health workers. Another negative point of REC that was reported by some health workers (2IDI, 2FGD) was the relative lack of treatment at the end of some consultations. When no cause of fever has been found, the REC posts a message asking the health worker to look for another cause of fever and to treat it, but no guidance is given on how to do so.

#### The “sick young infant aged up to 2 months” part of the algorithm

Some participants (2 IDI) identified the section dedicated to patients “under 2 months” as a weak part of the REC application. In IMCI, a temperature ≥ 37,5 °C in children under 2 months leads to the classification of “very severe disease” or “severe bacterial infection” which results in referral of the child. This need for referral was source of disagreement by health workers who felt it resulted in over-referral (2IDI, 1FGD).

#### HIV evaluation

Difficulties were reported in correctly performing HIV evaluation due to the perceived ambiguity of some of the clinical elements to be evaluated (IMCI includes several questions on the serological or virologic test results of the child and the mother, and then asks to look for the presence of 7 predictors of HIV infection) Table 4.

**Table 4:** Citations by participants related to dimension 3

Dimension 3: medical content of the algorithm	
Language or miscomprehension	
*“There are no difficult questions, but sometimes mothers have difficulties to understand. (…) You have to repeat to all of them and explain [what the question in REC is really requesting]. (…) For example, when the mother says: my child has diarrhoea; you have to ask her if there is blood in the stool. But some don’t know what this means, you have to insist: ‘when the child passes stool, exactly how do they look?’” Birth attendant, female*	
Missing pathologies	
*“We know that the diagnosis [in REC] comes from IMCI, but we see other pathologies that are not included in IMCI. If there is a possibility integrate other pathologies [into REC], it could help us to treat them. For example, when a child comes in with dermatitis, it is not included in IMCI, but it is still a consultation that you have to manage using REC” HO, male*	*“[In REC] there is no candidiasis, dysentery, wounds, burns, or conjunctivitis but we see a lot of them, it is very common (…) [It would be good] if we could add these to REC, as they are very frequent” RN, female*
Absence of final diagnosis	
*“We sometimes get children with fever who have a negative malaria RDT. You have to find the cause of the fever but the child has no diarrhoea, no cough… You don’t know which treatment to give because REC suggests none… (…) There is no diagnosis. (…) And I would like to be able to tell the mother: ‘this is what your child is suffering from.’*” *Birth attendant, female*	
Occasional lack of guidance in treatment	
*“If the fever is not elevated, REC offers no treatment at the end [of the consultation]. But the mother insists that the child had a fever and she came to you to present this issue. With [REC], one finds that the child has nothing, and you don’t know what to do…”* HO Male	
The “sick young infant aged up to 2 months” part of the algorithm	
*“Concerning new-borns, as soon as a new-born is febrile, when their temperature is higher than 37.5, REC tells you: severe bacterial infection and automatically advises you to refer the child. However, we sometimes assess [these patients] as cases we can and prefer to manage [ourselves, in the HF].” FGD*	*“What I find less good it is the part for children under 2 months. I feel there are cases we can manage at the health facility, but when you tick some signs on the device, REC tells you to refer…” Birth attendant, female*
HIV evaluation	
*“Here, in our context it is rare to see a child with an HIV test result, generally the mothers do it in the pre-natal consultations, but it is often not noted in the child’s health logbook. Therefore, it makes [this section of REC] a bit difficult. There are questions that are difficult to answer without proof and I find this part ambiguous.” RN male*	

### Dimension 4: medical knowledge on microbes, antimicrobials and resistance

When asked an open question about their knowledge on the different existing types of microbes, 9 participants (7IDI, 2FGD) spontaneously mentioned bacteria, but only 4 participants (3IDI, 1FGD) spontaneously mentioned virus, 3 of which were registered nurses (RN). Discussing the specific mechanisms of antibiotics, 5 participants (4IDI, 1FGD) correctly answered that antibiotics had no action on viral disease, 4 of which were RN. Advantages to prescribe an antibiotic mentioned by the participants were: treatment of fever and infection (6IDI, 1FGD), coverage of more serious disease (3IDI), faster or more complete healing (1IDI, 1FGD), prevention of exacerbation (1IDI). Risks or disadvantages of antibiotics were reported to be: toxicity - mostly mentioned as being induced by over dosage - (6IDI, 1FGD) such as kidney (4IDI) or hepatic injury (1IDI), allergies (2IDI, 1FGD) and gastro-intestinal toxicity such as perturbation of the gut flora or diarrhoea (2IDI, 1FGD). When talking about antibiotics, 6 participants (4IDI, 2FGD) spontaneously mentioned antibiotic resistance as being a negative consequence of antibiotic prescription. 5 (5IDI) came up with resistance or its concept when insisting on what could be long-term consequences of antibiotic over-prescriptions and 3 said they had heard about it but did not mention it neither spontaneously nor after orientation. Causes of resistance mentioned by the health workers were: under dosage of antibiotics (1FGD), abusive prescription (6IDI, 2FGD), prescription of a wide spectrum antibiotic although unnecessary (1IDI), lack of patients’ adherence to treatment (4IDI) or self-medication (2IDI, 1FGD) and repeated courses of antibiotics (1IDI).

### Dimension 5: impact of REC on patient management

#### The health worker perspective: better management and treatment

All participants reported that REC was beneficial to the management of children in at least one way. Positive effects were mentioned to be better management of children (5 IDI, 1FGD), facilitation in treatment decision-making and dosage calculation (7IDI, 1FGD), standardization of treatment (2IDI) and rational use of medicines (6IDI).

#### Additional prescription

The REC application guides the clinician trough the assessment of the child up to the treatment and the counselling part. At the end, a free text question gives room to the clinician to add additional classifications and treatments. Half of the study participants reported not to add an antibiotic when the REC application did not recommend it, and mentioned REC helped them to rationalize the use of drugs. However, the other half of the participants (6IDI, 1FGD) admitted to sometimes add an antibiotic even though REC did not recommend it. Reasons mentioned were: to calm or treat cough (5IDI), to prevent re-consultation (2IDI, 1FGD), to cover severe diseases or prevent worsening of the disease (3IDI) and in cases of fever with a negative malaria RDT result (1IDI, 1FGD). Some participants confessed not to feel confident in the malaria RDT result when the result was negative, especially when the child had no other cause of fever. However, they explained that those doubts were not making them add an antimicrobial but only asking the mother to come back with the child for a follow up visit.

#### Acceptance of home remedies and conservative treatments

REC recommends home remedies as conservative treatment such as infusion of honey, lemon and/or eucalyptus leaves for cough relief. Opinions regarding these remedies diverge: some health workers reported that, with explanation, home remedies were well accepted by caregivers, and noticed good outcomes of the child’s condition when using them. Other health workers reported the contextual difficulties of telling a mother (who had travelled to bring her child to the health facility) to just go home and prepare some tea. They also feared the dissatisfaction of caregivers, leading them to sometimes add an antibiotic treatment (Table 5).

**Table 5:** Citations by participants related to dimension 5

Dimension 5: Impact of REC on patient management	
The health worker perspective: better management and treatment	
*“For the prescription you do not need to look for medicines, it’s enough to just put the signs that the child has and REC will tell you the medicine indicated for the child. The other benefit is that the dosage is also given. Often it is difficult to calculate weight-adjusted dosages and you don’t know how much to give. But with REC, everything is inside, you just have to apply it.” Birth attendant, female*	*“The management of children, if it is applied correctly, is really good, it helps a lot. It also prevents us from prescribing drugs irrationally. (…) Often you could prescribe multiple substances to a child when they don’t need them. For instance, in a child with a cold we do not need to prescribe anything. REC tells you to do simple things, and in fact when the mother does them, it works out well.” HO, male*
*“The biggest advantage of REC, in my opinion, is that it helps us avoid a lot of deaths, especially in children. A second biggest advantage is that it prevents you hesitating over the treatment.” HO, male*	
Additional prescription	
*“When there is a history of fever, I add paracetamol. But if it is just a cough or cold, without fever or elevated respiratory rate, here we [can rather] explain to the mother how to make [supportive home remedies, such as] infusions of eucalyptus leaves or applying Shea butter in the nostrils of the child. We then tell them to come back in 2 days if the child is not better” RN, female*	*“For example, if a child comes in with a cough, and I see the child coughing really badly, but the respiratory rate is not elevated, personally, I add an antibiotic. Sometimes also if a child comes in with a fever of 39 °C or over and their RDT is negative (…) if the child has no other condition except for a cough, I add an antibiotic.” Birth attendant, female*
Confidence in malaria RDT	
*(Answering the question “do you trust the RDT result”)* “*Well… more or less… Often the child is really febrile, you perform the RDT and it is negative. The child has no other pathology that causes fever, they have no cough, no diarrhoea, no skin infection, they only have a fever. So we give a 2-day follow up appointment and tell the mother to bring the child back. We are obligated to see the child again, to re-evaluate if they have diarrhoea or cough that can cause fever, thus we only treat that” HO, female*	
Acceptance of home remedies and conservative treatments	
*“For a cough or cold, treatments are counselling [about home remedies/conservative treatments]. But yourself as a clinician in front of the patient, you feel the need to do something [else/more]. Because I have gained experience with such cases, (…) I feel the management [with home remedies] is not good, when you treat with antibiotics it works” HO, male*	*“You only have to explain to the mother, to lead her to understand that with cough or cold we do not need an antibiotic. The majority don’t argue, they tell you that you taught them something, like boiling eucalyptus leaves with some sugar” HO, female*
*“In our context it is difficult, when you see a mother who comes with her child coughing or with a cold, but the treatment is to tell her to make an infusion of eucalyptus leaves. Because in the health facility, the mother expects to receive drugs or products from the health centre, so she will leave unhappy if she now has to go find leaves and boil them. Because you know, here, we also have traditional practitioners prescribing leaves. The mothers don’t come to us for leaves. Sometimes we are obligated to give something else” RN, male*	

### Dimension 6: influence on health facility workflow and consultation organisation

#### Uptake of the REC by health workers

Overall, users mentioned having a good rate of uptake of the electronic algorithm and reported to use it in the management of nearly all children less than 5 years of age. Factors limiting the uptake were reported to be either 1) situational: emergencies (7IDI, 2FGD), reduced number of health workers (6IDI) or high attendance periods (8IDI, 2FGD); or 2) technical: a single device for several health workers (1IDI), bugs or breakdowns of the device or slowness of the navigation which increased waiting times and patient dissatisfaction (6IDI), especially during periods of high attendance. However, health workers reported making sure to organize themselves so that they can use the REC in all situations including in emergency situations or high patient flow periods.

### Impact on the health facility workflow

During the interviews, several health workers mentioned that the REC application contributed to positive changes in the HF organisation, such as: better organisation with task sharing and shifting, triage of children before the consultation and generation of systematic records of the child’s vital signs. Indeed, all HF visited during the study had specific organisation depending on the number of health workers available. If the later allowed it, often one was assigned to take the child’s vital signs before consultation. Some of the HF even trained a person from the community to measure these signs in order to improve the patient flow.

### REC impact on the consultation process

When asked about their knowledge on the normal course of a consultation, most of the health workers made a clear distinction between the history taking and the physical examination phase, that should be performed one after the other. The REC application has been programmed in a linear way, so that the health workers has to assess all signs related to a certain symptom before asking for the next symptom, which impedes them to take a full history followed by the entire physical examination. However, most of the participants reported to find the REC structure logical (9IDI, 1FGD); some mentioned that the REC structure was not following the normal consultation process but were now used to that way of performing consultation (2 IDI). Two clinicians (2IDI) reported however that the REC would be better if it could follow traditional consultation logic (Table 6).

**Table 6:** Citations by participants related to dimension 6

Dimension 6: Influence on the health facility workflow and consultation organisation	
Impact on the health facility workflow	
*“Here [in this health facility], when there are two health workers, [we have arranged that] one takes the vitals or performs the RDTs when needed [while the other performs the remaining steps of the REC consultation]. In high-attendance periods we try [to reorganise ourselves] to be at least two [for this purpose]” RN, female*	*“This morning we had two volunteers who came to help, so they take the height, brachial circumference and temperature and perform an RDT if the child has a fever. Only after that, do we start with REC, because you need to have all these data to start REC. (…) Before REC, we did not take those parameters so often” E10 Birth attendant, female*

### Dimension 7: health worker perception on training and supervision

#### REC as a learning tool and IMCI-REC training

The majority of users (10 IDI, 1FGD) reported that the electronic tool was increasing their knowledge, by making them learn. All health workers globally appreciated the training sessions on IMCI and the REC. The only complaint was about its relative shortness and density for those health workers who only received a 2 days training. The request of extending the training to the full 11 days for all health workers came up several times during the interviews.

#### Supervision visits by the district team and the “coaches”

As part of the IeDA project, HF supervision visits are conducted in average every 6 months. Feedbacks and comparison with the results of the other HF are then given to the clinician in charge of each HF. Participants talked positively about the evaluations, which make health worker want to improve, and make them reflect on the causes of the low scores. Trained coaches (members of the District Health Management team) also perform supervision visits in order to identify and address errors and support clinicians to deliver best practices. These visits were conducted every month during the first year of REC implementation and every 3 months thereafter and were globally perceived to be encouraging as they focused their attention on mistakes and helped them to learn and improve (Table 7).

**Table 7:** Citations by participants related to dimension 7

Dimension 7: Health worker perception on training and supervision	
REC as a learning tool	
*“REC is very important, every time you assess a child, some diagnoses and treatments will stay in your head. (…) It helps us to learn. If I am consulting a sick child [in a situation without REC], I will remember the signs: ‘I have already taken care of a child like this in REC it advised the following…’. It is a training (…), you’re learning while consulting, and even if REC is no longer in use, you [ex-REC users] would be able to treat children from 0 to 5 years more appropriately [due to this experience]” Birth attendant, female*	*“I’m happy because it trains us (…) right now if I am in a situation without REC, REC will stay in my head. (…)We know [by heart] the different paths you can take when a child has a cough, we know what to do until the end. There are questions we ask that other health workers would not. Not only do we apply IMCI but it stays in our head. With REC we have become used to doing things well from the beginning until the end every time” RN, male*

### Dimension 8: proposed improvement for future implementation

At the end of all interviews, an open question about what could be improved in the REC application was asked. Participants suggested more REC training sessions and the provision of certificates for this training. They also proposed financial compensations for using REC or receiving prizes for the best HF according to the evaluations, and that more frequent evaluations should take place. It was also recommended that REC extend its medical content to all age groups (adding older children, adolescents and adults), adding additional diagnostic classifications, but still keeping the application as simple as possible and fixing the slowness issues. Finally, some participants suggested that the home remedies recommended by REC should be provided by the HF and that REC should expand its implementation to all districts of Burkina Faso.

## Discussion

This study investigating health worker perceptions on the use of the REC application in Burkina Faso revealed a high degree of acceptance of the electronic device. All participants expressed their general satisfaction in using the tool. Beyond the effect on the consultation itself, they had all observed major (and also several interesting minor) impacts on various aspects of the healthcare system. There was a clear demand, not only from the health workers but also from the children’s guardians and members of their communities, for expansion of the temporal, demographic and geographic scope of the intervention.

### Electronic, technical aspects and the human-tablet interface

Participants felt confident using an eCDA on tablet to guide them during consultation. Not only did they report feeling comfortable in terms of device usability and navigation but they also reported to be incentivised by a personal pride of using a new technology. All participants had previous personal or professional IT experience, which helped them learn and adapt to the tablet more rapidly. Interestingly, in a Tanzanian study on eIMCI integrated into PDAs in 2012, where participants had little IT experience, use of the device was not reported to be an issue [11]. The device was perceived as a powerful tool guiding clinicians through a structured consultation, that better ensured more complete and higher quality evaluations of the child [11]. Participants reported that the eCDA use did not limit their relationship with the patient and was rather met with general satisfaction by caretakers and their families. Our direct observations confirmed this high level of acceptance amongst patients and the community and revealed strong desire for sustainability of the intervention. This was probably also due to the considerable effort undertaken by the team of the IeDA program to reinforce communication and community outreach in order to inform the local public about the intervention.

### Slowness of the device and consultation durations

Slowness (that was due to the high number of patient files saturating the memory of the device) was highlighted as being an important inconvenience of the eCDA, discouraging its use during high-attendance periods. Participants however concluded that REC could increase consultation duration compared to routine practice but reduced it compared to a paper-IMCI consultation. Findings from the Mitchel et al. study showed that PDAs were faster to use than paper formats of IMCI [11], while Shao et al. reported having observed a slight increase in consultation time compared to another study on paper IMCI [12, 15]. The benefits of adding more topics or components to the algorithm should thus be balanced with the risk of increasing the length of the consultation, which may discourage clinicians to use the tool. Technical solutions to reduce slowness of the tablets are also essential.

### Duplication of work in parallel systems

Participants reported dissatisfaction about the duplication of work when paper and electronic systems remained running in parallel. Shao and al. also highlighted similar complaints [12]. In order to enable sustainable up take of eCDA based interventions, strong efforts should thus be made to align diagnostic and treatment classifications and fully integrate the new intervention into the existing system, so that paper-based data collection can be efficiently replaced as soon as possible, and rather provide an incentive for uptake.

### Frequent diagnostic classifications missing in the algorithm

As the REC application is an IMCI derived algorithm, some of its strengths and limits are directly due to the content of IMCI itself. Others are related to the way the IMCI guidelines have been interpreted when creating the electronic version; indeed, a paper version does not provide a recommendation for each possible combination of clinical elements, while an electronic version is obliged to do so.

Participants expressed a strong desire for getting guidance not only on serious diseases (which has always been the focus of IMCI, its main aim being to decrease mortality) but also on the management of frequent diseases (such as rhinitis, urinary tract infection, dermatitis, or burns and wounds). The net result is the lack of guidance on the final diagnostic classification and treatment to give for a substantial number of patients. This was a source of frustration for them, not only because many patients could not benefit from the tool but also because they are obligated to use the REC despite its lack of help for those pathologies. Frequent paediatric diseases should thus be added to the REC, which would not increase the consultation duration as most patients have only one acute diagnostic classification at a time. Another demand was to extend the REC to children older than 5 years, adolescents and adults as they have to provide care to the entire population that could then benefit from higher quality care.

### Lack of guidance for management of fever without focus

Health workers complained about the lack of guidance when no specific cause of fever could be found. There is strong evidence that viral infections are responsible for a large proportion of fevers in children [16] and that no antibiotics are required in most children presenting with fever without focus [17]. Open-ending algorithm pathways which conclude with vague recommendations such as “look for other causes of fever”, as well as “negative diagnoses” such as “Fever - no malaria” were source of confusion and should be avoided. Negative classifications should be replaced by positive diagnoses, even if presumptive, such as “probable viral infection”, and a specific management should be proposed, such as lowering temperature and explaining to caretakers that the disease is generally self-limited, that taking antibiotic would bring no benefit and to come back in case of deterioration.

### Knowledge and perception on antimicrobial prescription and resistance

When asked about existing types of microbes, viruses were cited by only 30% of participants. Three-quarters of the participants who spontaneously mentioned virus were RNs (as were those knowing antibiotics’ action and risk of resistance), highlighting the importance of the level of education. If eCDAs have strong potential for reducing unnecessary antibiotic prescription, as showed in a recent study [13], it is essential that health workers adhere to the recommendations and do not prescribe antimicrobials unnecessarily. Knowledge on the high prevalence of viruses and inefficacity of antibiotics to treat viral diseases should be raised, during both pre-graduate and in-service training. Pop-up messages recalling reasons why antibiotics are not recommended in that particular patient could also decrease the clinician’s temptation to prescribe.

Home remedies and conservative therapy or watchful waiting, that are often recommended, were however reported to be poorly accepted by caretakers who were disappointed to not have received a medicine. Mitchell et al. highlighted that treatments were harder to carry out if they were contrary to the patient’s expectations [11]. Preparation of local home remedies at the HF in advance could potentially be a solution to increase acceptance from the child’s guardian, as proposed by several study participants.

### Workflow at the health facility

The implementation of the eCDA REC not only improved the consultation quality but also changed the whole HF organisation. It promoted task-sharing and -shifting through the systematic record of the child’s vital signs (temperature, respiratory rate, and weight) before the consultation by a less trained health worker. This shift is an opportunity to strengthen children’ triage upon arrival, to identify those who should be referred immediately to a higher level of care.

### Limitations

This study was focused on primary health workers working in rural regions of Burkina Faso where the IeDA intervention had been deployed. Results may not be generalisable to urban settings, where number of health workers, patients, and HF infrastructure is usually different. Another limitation is that this study represents a snapshot taken at a particular time in a program that is constantly improving. Indeed, both the tool itself and the implementation process, undergo a robust monitoring and evaluation process aiming to find and address negative issues. The described problems have thus already been addressed by the IeDA team. Finally, this study was conducted through health workers with varying types of professional backgrounds. While the IMCI strategy in Burkina Faso targets nurses, health worker with less training might not fully understand the IMCI content, strategy and philosophy and their opinion might thus have been biased by this fact.

## Conclusions

This qualitative study revealed key insights on the primary health care worker’s perspective on the use of eCDAs. Findings showed that the use of an electronic device to guide the clinician’s daily practice might have potentially positive impacts on several aspects of the health care system, which go beyond the quality of the care and the decrease of unnecessary antibiotic prescriptions. While general satisfaction was reported by the users, efforts have to be made to optimize the algorithm’s medical content and to improve the integration of these devices in the health system in order to reduce barriers to its utilisation and enable sustainable uptake. In addition to improvement of the of quality of care, eCDAs open the possibility of future tools that may provide guideline based targeted supervision of health workers, help manage medicines and stock supply, provide monitoring and evaluation of health care interventions and improve epidemiological surveillance.

## Additional file

Additional file 1: Burkina Faso’s health profile:description of the Burkina Faso’s health profile, including population, maternal mortality, children mortality, main causes of death and immunization rate. Burkina Faso’s health system organization:description of the Burkina Faso’s health system organization, different level of health care, activities and funding. (DOCX 78 kb)

## Abbreviations

BA: Birth attendant (Accoucheuse)
CSPS: Centre de santé et de prévention sociale, (primary health care facilities)
eCDA: electronic Clinical Decision Algorithms
eIMCI: electronic Integrated Management of Childhood Illness
FGD: Focus group discussions
HF: Health facilities
HO: Health officer (Agent itinérant de santé)
IDI: In-depths interviews
IeDA: Integrated electronic Diagnostic Approach
IMCI: Integrated Management of Childhood Illness
LN: Licensed nurse (Infirmier breveté)
MW: Midwife (Sage-femme)
NGO: Non governmental organization
PDA: Personal digital assistant
RDT: Rapid diagnostic test
REC: Register of Consultation (in french registre électronique des consultations)
RN: Registered nurse (Infirmier diplomé d’état)
Tdh: Terre des hommes
UNICEF: United Nations International Children’s Emergency Fund
WHO: World Heath Organisation
