# Supplementary material for: Large-scale implementation of electronic Integrated Management of Childhood Illness (eIMCI) at the primary care level in Burkina Faso: a qualitative study on health worker perception of its medical content, usability and impact on antibiotic prescription and resistance
Source: BMC Public Health. 2019 Apr 29;19:449. doi: 10.1186/s12889-019-6692-6 (PMC6489291; doi:10.1186/s12889-019-6692-6)
Supplement: Supplementary file 1 — Burkina Faso’s health profile:description of the Burkina Faso’s health profile, including population, maternal mortality, children mortality, main causes of death and immunization rate. Burkina Faso’s health system organization:description of the Burkina Faso’s health system organization, different level of health care, activities and funding. (DOCX 78 kb) [file 12889_2019_6692_MOESM1_ESM.docx]

**Appendix**

**Study context**

**Burkina Faso’s health profile**

According to the global health observatory data repository from WHO, the population of Burkina Faso exceeded 18 million in 2015, with 46% of people aged less than 15-years-old. The maternal mortality rate was of 371 for 100,000 live births. 54 children out of 1000 would die before the age of 1 year and 88 before the age of 5 years. Main causes of death among children were malaria, acute lower respiratory infections and other perinatal and nutritional conditions. Immunization rate for the first dose of the measles vaccine reached 88% among one-year olds and 50% for the second dose by the recommended age.

**Burkina Faso’s health system organization**

Burkina Faso’s health system is organized in different levels providing primary, secondary and tertiary care. The first level of care consists of the “centre de santé et de promotion sociale” (CSPS) which are health and social promotion centres. Depending on their size, they accommodate one or more health workers and usually have a consultation room, a procedure room, a delivery room and a pharmacy. They receive a high number of patients especially during the season of high transmission of malaria and provide diverse activities, ranging from curative care of common diseases to management of malnutrition, vaccination and sensitization of the population on common nutrition or health problems. Since May 2016, Burkina Faso has benefited from the implementation of free healthcare for children under 5 and pregnant women.
